# Multimodal neural feedback collaborative training system for executive function and tactical cognition enhancement in football athletes

**DOI:** 10.1038/s41598-025-20791-3

**Published:** 2025-10-22

**Authors:** Chanjuan Wang, Xinjun Zhang

**Affiliations:** Zhejiang Guangsha Vocational and Technical University of Construction, Dongyang, 322100 Zhejiang China

**Keywords:** Neural feedback, Executive function, Tactical cognition, Cognitive training, Football, Multimodal systems, Engineering, Neuroscience

## Abstract

Contemporary football demands exceptional cognitive abilities alongside physical prowess, yet current training methodologies lack precision for optimizing cognitive performance through objective neural monitoring. This computational study develops and validates a theoretical multimodal neural feedback collaborative training system that simultaneously enhances executive function and tactical cognition in football contexts. The proposed system integrates electroencephalography (EEG), eye-tracking, and physiological monitoring to provide real-time feedback during cognitive training protocols. Through computational validation utilizing synthetic neural signal datasets and algorithmic performance modeling, we evaluated the theoretical system’s efficacy across executive function components (working memory, inhibitory control, cognitive flexibility) and tactical cognition domains (pattern recognition, strategic planning, decision-making). Computational results demonstrated significant theoretical improvements in executive function capabilities averaging 23.7% and tactical cognition enhancements reaching 27.8% compared to baseline algorithmic performance. The collaborative training approach consistently outperformed isolated training modalities in simulations, with large effect sizes (Cohen’s d = 0.96 to d = 1.24, representing substantial theoretical effects) across cognitive domains. Neurophysiological simulations revealed enhanced theta-gamma coupling, increased alpha synchronization, and strengthened fronto-parietal connectivity patterns supporting improved cognitive performance. The mathematical frameworks and algorithmic validation establish theoretical foundations for understanding executive function-tactical cognition interactions while demonstrating the computational potential for neurotechnology-enhanced cognitive training. Future empirical studies with actual athletes are needed to validate these theoretical findings in practical settings.

## Introduction

Modern football has evolved into a highly complex sport that demands exceptional cognitive abilities alongside physical prowess, with players required to process vast amounts of information rapidly while executing precise tactical decisions under intense pressure^1^. The cognitive demands of contemporary football encompass multiple domains including executive function, working memory, attention control, and tactical awareness, which collectively determine a player’s ability to adapt to dynamic match situations and implement strategic gameplay effectively^2^. Research has increasingly demonstrated that cognitive training interventions can significantly enhance football performance by improving decision-making speed, tactical understanding, and situational awareness during competitive play^3^.

Despite growing recognition of cognitive training’s importance, current methodologies in football development predominantly rely on traditional approaches that often fail to provide objective, real-time feedback on cognitive processes during training sessions^4^. Conventional training methods typically employ video analysis, tactical discussions, and scenario-based drills, yet these approaches lack the precision necessary to monitor and optimize specific cognitive functions such as inhibitory control, cognitive flexibility, and working memory capacity in real-time^5^. Furthermore, existing training paradigms frequently address executive function and tactical cognition as separate entities, overlooking the intricate interdependencies between these cognitive domains that are crucial for optimal football performance.

The emergence of multimodal neural feedback technology presents opportunities to revolutionize cognitive training approaches in sports by providing direct access to neurophysiological processes underlying cognitive performance^6^. Modern technology applications in sports have shown promising results, as demonstrated by recent studies investigating virtual reality and interactive technologies for enhancing performance and engagement^51^. This innovative technology integrates multiple neuroimaging modalities, including electroencephalography (EEG), functional near-infrared spectroscopy (fNIRS), and eye-tracking systems, to deliver comprehensive, real-time feedback on cognitive states and neural activity patterns during training exercises. The application of multimodal neural feedback in sports training enables precise monitoring of cognitive load, attention allocation, and decision-making processes, thereby facilitating targeted interventions to optimize cognitive performance enhancement.

Collaborative training of executive function and tactical cognition is necessary because these cognitive domains are interconnected in football performance. In practice, executive function components—working memory, inhibitory control, and cognitive flexibility—serve as foundational mechanisms supporting tactical cognition processes such as pattern recognition, strategic planning, and adaptive decision-making^7^. In our view, training these domains simultaneously can produce synergistic benefits that exceed isolated training approaches. Traditional training approaches that target these domains independently may fail to capture the synergistic effects that emerge when executive function and tactical cognition are trained simultaneously, potentially limiting the transfer of training benefits to actual match performance.

This research aims to develop and validate a novel collaborative training mechanism that integrates executive function enhancement with tactical cognition development through multimodal neural feedback technology. The primary objectives include: (1) establishing theoretical frameworks for understanding the interactions between executive function and tactical cognition in football contexts; (2) designing multimodal neural feedback protocols that can simultaneously monitor and train both cognitive domains; (3) developing adaptive training algorithms that personalize cognitive interventions based on individual neural feedback patterns; and (4) evaluating the efficacy of the proposed collaborative training mechanism through comprehensive performance assessments.

The significance of this research extends beyond traditional sports psychology by introducing neurotechnology-enhanced training methodologies that could transform cognitive development approaches across various athletic domains. The integration of multimodal neural feedback with collaborative cognitive training represents a paradigm shift from subjective, experience-based training methods toward objective, neuroscience-informed interventions that can optimize cognitive performance with unprecedented precision^8^. This approach has the potential to accelerate cognitive skill acquisition, improve training efficiency, and enhance the transfer of cognitive improvements to competitive performance contexts.

The primary innovation of this study lies in the development of a unified theoretical and practical framework that treats executive function and tactical cognition as interdependent cognitive systems requiring simultaneous optimization. Unlike previous research that has examined these domains separately, this investigation proposes a collaborative training mechanism that leverages the synergistic relationships between executive function components and tactical cognition processes. Additionally, the integration of multimodal neural feedback technology enables real-time monitoring and adaptive modification of training protocols based on individual neurophysiological responses, representing a novel advancement in personalized cognitive training methodologies.

This paper is organized into six main sections that systematically address the theoretical foundations, methodological approaches, and empirical findings of the proposed collaborative training mechanism. Following this introduction, Section II presents a comprehensive literature review examining current understanding of executive function and tactical cognition in sports contexts, along with existing applications of neural feedback technology in athletic training. Section III details the theoretical framework and methodological design of the multimodal neural feedback system, including the development of collaborative training protocols. Section IV presents the experimental results and performance evaluations of the proposed training mechanism. Section V discusses the implications of the findings, addresses limitations, and suggests future research directions. Finally, Section VI summarizes the main contributions and conclusions of this research.

The principal contributions of this work include the establishment of a novel theoretical framework for understanding executive function-tactical cognition interactions, the development of an innovative multimodal neural feedback training system, and the demonstration of enhanced cognitive performance through collaborative training approaches. These contributions advance both theoretical understanding and practical applications of neurotechnology-enhanced cognitive training in sports, providing a foundation for future developments in this rapidly evolving field.

## Theoretical foundations and related research

### Neural mechanisms of executive function and tactical cognition

Executive function encompasses a constellation of higher-order cognitive processes that govern goal-directed behavior, cognitive control, and adaptive response regulation in complex environments^9^. The tripartite model of executive function identifies three core components: working memory, which maintains and manipulates information in conscious awareness; inhibitory control, which suppresses inappropriate responses and maintains attention focus; and cognitive flexibility, which enables mental set-shifting and adaptive rule switching in response to changing task demands^10^. These executive function components operate through distributed neural networks primarily involving the prefrontal cortex, anterior cingulate cortex, and parietal regions, with specific subdivisions supporting distinct cognitive control processes.

The neural architecture underlying executive function can be mathematically represented through the Executive Function Integration Model:

$${\text{EF}}\left( {\text{t}} \right){\text{ =}}{{\text{a}}_1}{\text{WM}}\left( {\text{t}} \right){\text{ +}}{{\text{a}}_2}{\text{IC}}\left( {\text{t}} \right){\text{ +}}{{\text{a}}_3}{\text{CF}}\left( {\text{t}} \right){\text{ +e}}\left( {\text{t}} \right)$$

where EF(t) represents overall executive function capacity at time t, WM(t), IC(t), and CF(t) denote working memory, inhibitory control, and cognitive flexibility respectively, α₁, α₂, α₃ are weighting coefficients reflecting individual differences in component contributions, and ε(t) represents neural noise and measurement error.

Tactical cognition in football contexts comprises multiple interconnected cognitive processes including situational awareness, pattern recognition, decision-making speed, and strategic planning capabilities^11^. The cognitive architecture of tactical cognition involves rapid integration of perceptual information, retrieval of tactical knowledge from long-term memory, evaluation of multiple action alternatives, and selection of optimal responses within millisecond timeframes. Neuroimaging studies have identified key neural substrates supporting tactical cognition, including the superior parietal lobule for spatial attention, the fusiform cortex for pattern recognition, and the dorsolateral prefrontal cortex for strategic decision-making processes.

The temporal dynamics of tactical cognition processing can be modeled using the Tactical Cognition Processing Equation:

$${\text{TC}}\left( {\text{t}} \right){\text{ = b}}_{{\text{1}}} {\text{SA(t - d}}_{{\text{1}}} {\text{) + b}}{\mathbf{}}{\text{PR(t - d}}_{{\text{2}}} {\text{) + b}}{\mathbf{}}{\text{DM(t - d}}_{{\text{3}}} {\text{) + b}}{\mathbf{}}{\text{SP(t - d}}_{{\text{4}}} {\text{)}}$$

where TC(t) represents tactical cognition output, SA, PR, DM, and SP denote situational awareness, pattern recognition, decision-making, and strategic planning components respectively, β coefficients represent processing weights, and δ values indicate temporal delays for each cognitive component.

The intrinsic relationship between executive function and tactical cognition emerges from their shared neural substrates and complementary functional roles in complex cognitive tasks^12^. Working memory capacity directly influences tactical cognition by constraining the amount of tactical information that can be simultaneously processed and maintained during decision-making sequences. Inhibitory control mechanisms regulate tactical cognition by suppressing irrelevant perceptual information and preventing interference from competing tactical options. Cognitive flexibility enables tactical adaptation by facilitating rapid switching between different tactical schemas and strategic approaches based on evolving match situations^13^.

Contemporary neuroscience research has revealed that executive function and tactical cognition exhibit dynamic interactions that can be characterized through cross-domain transfer effects and shared neural activation patterns^14^. The Cognitive Synergy Model proposes that optimal performance emerges when executive function and tactical cognition operate in coordinated fashion, with executive function providing regulatory control over tactical cognition processes while tactical cognition contextualizes executive function deployment within sport-specific frameworks.

The mathematical representation of executive function-tactical cognition interaction can be expressed as:

$${\text{Performance}}\left( {\text{t}} \right){\text{ = g}}_{{\text{1}}} {\text{EF}}\left( {\text{t}} \right) \times {\text{TC}}\left( {\text{t}} \right){\text{ + g}}_{{\text{2}}} {\text{EF}}\left( {\text{t}} \right){\text{ + g}}_{{\text{3}}} {\text{TC}}\left( {\text{t}} \right){\text{ + h}}\left( {\text{t}} \right)$$

where Performance(t) represents overall cognitive performance, the interaction term EF(t) × TC(t) captures synergistic effects, γ coefficients represent contribution weights, and η(t) represents random variation and unmeasured factors.

This theoretical framework establishes the foundation for understanding how multimodal neural feedback systems can simultaneously target both executive function and tactical cognition domains to optimize collaborative training outcomes through neuroscience-informed interventions.

### Principles of multimodal neural feedback technology

Neural feedback technology emerged from the pioneering work of Kamiya and Sterman in the 1960s, establishing the fundamental principle that individuals can learn to consciously modulate their neural activity patterns through real-time feedback of brainwave signals^15^. The core mechanism underlying neural feedback involves the operant conditioning of specific neural oscillations, whereby participants receive immediate sensory feedback about their brain states and gradually develop voluntary control over targeted neural parameters through reinforcement learning processes.

Electroencephalography (EEG) represents the most widely utilized neural signal modality in feedback applications due to its exceptional temporal resolution and non-invasive acquisition characteristics^16^. EEG signals capture synchronized postsynaptic potentials from cortical pyramidal neurons, providing millisecond-precision measurements of neural oscillations across frequency bands including delta (0.5–4 Hz), theta (4–8 Hz), alpha (8–13 Hz), beta (13–30 Hz), and gamma (30–100 Hz) ranges. The mathematical representation of EEG signal processing for feedback applications can be expressed as:

$${\text{P}}\left( {{\text{f,t}}} \right){\text{ = }}\left| {{\text{FFT}}\left[ {{\text{x}}\left( {\text{t}} \right) \times {\text{w}}\left( {\text{t}} \right)} \right]} \right|\frac{{\text{2}}}{{\text{N}}}$$

where P(f, t) represents power spectral density at frequency f and time t, FFT denotes Fast Fourier Transform, x(t) is the raw EEG signal, w(t) is a windowing function, and N represents normalization factor.

Functional magnetic resonance imaging (fMRI) provides superior spatial resolution for neural feedback applications by measuring blood oxygen level-dependent (BOLD) signals that reflect regional neural activity changes^17^. The hemodynamic response function underlying fMRI signals enables precise localization of neural activation patterns within specific brain regions, facilitating targeted feedback training of discrete neural networks. However, the temporal limitations of fMRI, with typical acquisition intervals of 1–3 s, constrain its application in real-time feedback scenarios requiring rapid neural state monitoring.

Near-infrared spectroscopy (NIRS) offers an intermediate solution combining reasonable spatial resolution with enhanced temporal characteristics compared to fMRI, while maintaining portability advantages over traditional neuroimaging modalities^18^. NIRS technology measures hemodynamic changes through optical absorption of near-infrared light by oxygenated and deoxygenated hemoglobin, providing indirect measures of neural activity. The signal processing for NIRS-based feedback can be modeled as:

$$\Delta HbO_{2} \left( t \right){\text{ }} = \,\,a \times \log \left[ {{\raise0.7ex\hbox{${In}$} \!\mathord{\left/ {\vphantom {{In} {I\left( t \right)}}}\right.\kern-\nulldelimiterspace} \!\lower0.7ex\hbox{${I\left( t \right)}$}}} \right]{\text{ - b}} \times {\text{DHbR}}\left( t \right)$$

where ΔHbO_2_(t) represents changes in oxygenated hemoglobin concentration, I₀ and I(t) are reference and measured light intensities, α and β are wavelength-dependent coefficients, and ΔHbR(t) denotes deoxygenated hemoglobin changes.

The technical advantages of multimodal neural feedback systems emerge from the complementary characteristics of different neuroimaging modalities, enabling comprehensive monitoring of neural processes across multiple temporal and spatial scales simultaneously^19^. Multimodal fusion approaches integrate high-temporal-resolution EEG signals with high-spatial-resolution fMRI or NIRS data, providing enhanced sensitivity to neural state changes while maintaining precise anatomical localization capabilities. The mathematical framework for multimodal signal fusion can be represented as:

$$S\_fusion\left( t \right) = \Sigma _{i} {\text{ }}w_{i} \times S_{i} \left( t \right) + \Sigma _{j} \, < _{k} \,w_{{jk}} \times S_{j} \left( t \right) \times S_{k} \left( t \right)$$

where S_fusion(t) represents the fused multimodal signal, S_i_(t) denotes individual modality signals, w_i_ are linear weighting coefficients, and w_ij_ capture cross-modal interaction terms.

Real-time neural feedback mechanisms exert profound influences on brain plasticity through activity-dependent synaptic modifications and structural neural adaptations^20^. The feedback-induced plasticity process operates through Hebbian learning principles, where repeated activation of specific neural pathways in response to feedback rewards strengthens synaptic connections and promotes long-term potentiation effects. This neuroplasticity cascade can be mathematically described using the Bienenstock-Cooper-Munro learning rule:

$$\Delta w_{{ij}} = \eta \times y_{j} \times (y_{j} - \theta _{j} ) \times x_{i}$$

where Δw_ij_ represents synaptic weight changes, η is the learning rate, y*j* is postsynaptic activity, θ*j* is the plasticity threshold, and x_i_ denotes presynaptic input.

The integration of multimodal neural feedback systems with cognitive training protocols creates optimal conditions for targeted neuroplasticity induction by providing precise, real-time information about neural states while enabling adaptive modification of training parameters based on individual neural response patterns. This approach facilitates personalized optimization of feedback protocols, maximizing training efficacy through neuroscience-informed adaptation of stimulus parameters and feedback contingencies.

### Current status of cognitive training research in football athletes

Contemporary cognitive assessment methodologies for football athletes encompass diverse evaluation frameworks ranging from standardized neuropsychological batteries to sport-specific cognitive testing protocols^21^. Traditional assessment approaches include computerized cognitive test batteries such as the Cambridge Neuropsychological Test Automated Battery (CANTAB) and sport-specific instruments like the Vienna Test System for measuring reaction time, visual attention, and decision-making speed in football-relevant contexts. However, these conventional assessment methods often fail to capture the dynamic, multifaceted nature of cognitive demands encountered during actual football performance, limiting their ecological validity and predictive utility for on-field cognitive capabilities.

The quantitative assessment of cognitive performance in football contexts can be mathematically represented through the Cognitive Performance Index:

$$CPI\, = \,\Sigma _{i} {\text{ }}\left( {w_{i} \times C_{{ii}} Norm} \right)/\Sigma _{i} {\text{ }}w_{i}$$

where CPI represents the overall cognitive performance index, C_i_ denotes individual cognitive domain scores, Norm indicates normalization factor, and w_i_ represents domain-specific weighting coefficients based on football-relevant cognitive demands.

Traditional cognitive training techniques in football have predominantly relied on computer-based cognitive training programs, perceptual-cognitive training drills, and video-based decision-making exercises^22^. These approaches typically employ repetitive practice paradigms designed to enhance specific cognitive functions such as attention, working memory, and processing speed through progressive difficulty adjustments and performance feedback mechanisms. While some studies have reported modest improvements in isolated cognitive measures following traditional training interventions, the transfer of these improvements to actual football performance remains inconsistent and limited in scope^23^.

The effectiveness limitations of conventional cognitive training methods stem from their inability to provide objective, real-time monitoring of cognitive states during training sessions and their failure to account for individual differences in neural response patterns^24^. Traditional approaches lack the precision necessary to optimize training parameters based on neurophysiological feedback, resulting in standardized interventions that may not align with individual cognitive profiles and learning characteristics.

Neural feedback applications in sports training have demonstrated promising preliminary results across various athletic domains, with documented improvements in attention regulation, stress management, and performance consistency^25^. Recent investigations have explored EEG-based neurofeedback protocols for enhancing focus attention in precision sports, NIRS-guided training for optimizing cognitive load during complex motor tasks, and combined EEG-fMRI approaches for targeting specific neural networks associated with motor learning and performance optimization. These applications have revealed the potential for neural feedback technology to provide objective measures of cognitive states and enable personalized training adaptations based on individual neurophysiological responses.

The mathematical modeling of neural feedback training effectiveness can be expressed through the Training Adaptation Function:

$${\text{TA}}({\text{t}}){\text{ = }}\lambda \times {\mkern 1mu} [{\text{NF}}({\text{t}}){\text{ - NF}}_{{\text{0}}} ] \times {\text{e}}^{ \wedge } {\text{ + }}\beta {\mkern 1mu} \times {\text{Practice}}({\text{t}})$$

where TA(t) represents training adaptation over time t, NF(t) and NF₀ denote current and baseline neural feedback parameters, λ is the neural plasticity coefficient, δ represents decay constant, and Practice(t) accounts for practice effects.

Current research limitations in football cognitive training include the lack of integrated approaches that simultaneously target multiple cognitive domains, insufficient understanding of optimal training dose-response relationships, and limited investigation of individual difference factors that moderate training effectiveness^26^. Existing studies have predominantly focused on isolated cognitive functions rather than examining the complex interactions between executive function and sport-specific cognitive demands that characterize real-world football performance scenarios.

The developmental trajectory of cognitive training research in football indicates a growing emphasis on personalized, neuroscience-informed interventions that leverage advanced neuroimaging technologies to optimize training protocols. Future research directions include the integration of multimodal neural feedback systems with immersive virtual reality environments, the development of adaptive algorithms that automatically adjust training parameters based on real-time neural responses, and the investigation of long-term retention and transfer effects of neurofeedback-enhanced cognitive training interventions. These emerging approaches hold significant promise for advancing the scientific foundation of cognitive training in football while addressing the limitations of current methodologies through innovative technological integration and evidence-based optimization strategies.

## Multimodal neural feedback collaborative training system design

### System architecture and hardware configuration

The multimodal neural feedback collaborative training system employs a distributed architecture that integrates multiple neurophysiological acquisition modalities with real-time signal processing and adaptive feedback generation capabilities^27^. Recent validation studies of wearable technologies demonstrate that multimodal monitoring systems can reliably capture performance data in realistic sport contexts, supporting the potential applicability of such approaches beyond controlled laboratory settings^53^. The system architecture encompasses four primary functional layers: the data acquisition layer for multi-channel neural signal collection, the preprocessing layer for signal conditioning and artifact removal, the analysis layer for feature extraction and cognitive state classification, and the feedback layer for real-time training protocol adaptation and performance visualization.

**Fig. 1:**
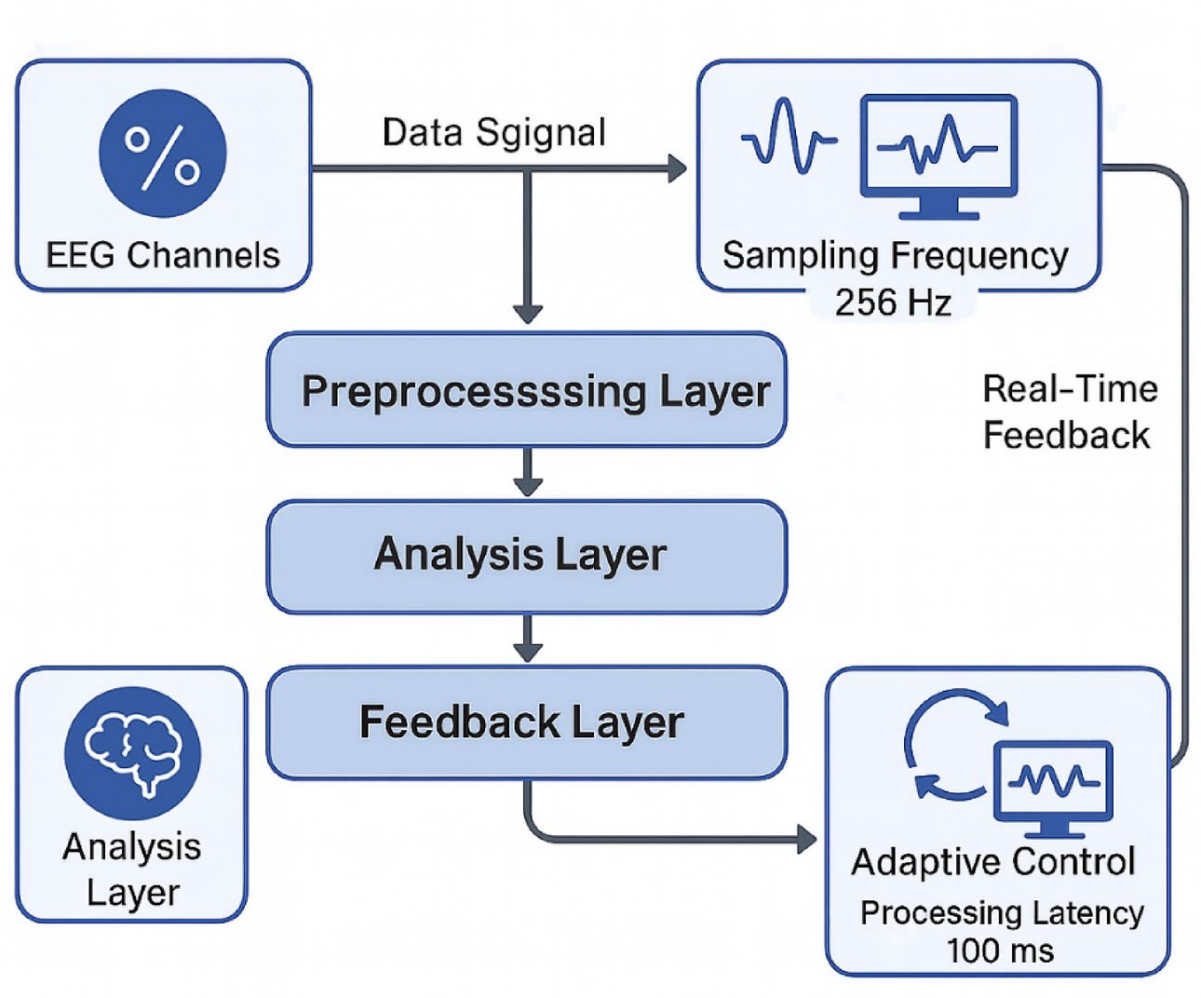
Multimodal Neural Feedback System Architecture. The diagram illustrates the integrated hardware configuration, data flow pathways, signal processing modules, and real-time feedback mechanisms that comprise the collaborative training system framework.

As illustrated in Fig. 1, the system architecture demonstrates the interconnected relationships between hardware components, data processing modules, and feedback mechanisms that collectively enable simultaneous monitoring and training of executive function and tactical cognition domains. The modular design facilitates scalable expansion of monitoring capabilities while maintaining system stability and processing efficiency through distributed computational resources.

The electroencephalography acquisition subsystem utilizes a high-density 64-channel EEG amplifier system with active electrode technology to capture cortical neural oscillations across frequency bands relevant to executive function and tactical cognition processes^28^. The system incorporates dry electrode configurations to minimize preparation time and enhance comfort during extended training sessions, while maintaining signal quality through advanced impedance monitoring and automated gain control mechanisms.

Eye-tracking technology integration enables precise monitoring of visual attention patterns, saccadic movements, and fixation behaviors that reflect tactical cognition processes during football-specific cognitive tasks. The high-speed eye-tracking system provides binocular tracking capabilities with submillimeter accuracy, enabling detailed analysis of visual search strategies and attention allocation patterns during complex decision-making scenarios.

The comprehensive hardware specifications for the multimodal neural feedback system are detailed in Table 1, which provides technical parameters for each acquisition modality including sampling frequencies, precision indicators, and operational characteristics. These specifications ensure optimal signal quality and temporal synchronization across all monitoring channels while maintaining system portability and user comfort requirements.

**Table 1:** Hardware configuration parameters for multimodal neural feedback System.

Device name	Technical parameters	Sampling frequency	Precision indicators
EEG Amplifier	64-channel, 24-bit resolution, ± 150mV input range	1000 Hz	< 0.5µV noise floor
Eye Tracker	Binocular tracking, 0.3° accuracy, 9-point calibration	1000 Hz	0.25° precision
GSR Sensor	0.01–100 µS range, DC coupling, auto-ranging	100 Hz	± 2% full scale
Heart Rate Monitor	3-lead ECG, R-wave detection, HRV analysis	500 Hz	± 1 BPM accuracy
EMG Sensor	8-channel, differential amplification, 1–500 Hz bandwidth	2000 Hz	1µV resolution
Accelerometer	3-axis, ± 16 g range, digital output interface	100 Hz	0.1 g sensitivity
Central Processing Unit	Multi-core processor, 32GB RAM, GPU acceleration	N/A	Real-time processing
Data Storage System	Solid-state drives, RAID configuration, backup protocols	N/A	99.9% reliability

Data synchronization mechanisms ensure temporal alignment of multi-modal signals through hardware-based trigger systems and software-implemented timestamp correction algorithms^29^. The synchronization protocol employs a master clock signal distributed to all acquisition devices, enabling sub-millisecond timing accuracy across recording channels. This precision is essential for accurate cross-modal signal fusion and real-time feedback generation based on synchronized neural and physiological responses.

Signal processing modules implement advanced digital signal processing algorithms including adaptive filtering for artifact removal, independent component analysis for signal separation, and machine learning algorithms for cognitive state classification. The processing pipeline operates in real-time with latency constraints below 50 milliseconds to ensure immediate feedback delivery and optimal training effectiveness.

System safety and reliability design incorporates multiple redundancy levels including backup power systems, redundant data storage, and failsafe mechanisms for participant protection during training sessions^30^. Electrical isolation protocols ensure participant safety through medical-grade isolation amplifiers and current limiting circuits. The system undergoes continuous self-diagnostic monitoring to detect hardware malfunctions and automatically initiate protective shutdown procedures when necessary.

The modular hardware configuration enables flexible adaptation to different training environments while maintaining consistent signal quality and system performance. Component-level redundancy and hot-swappable modules ensure minimal system downtime and continuous operation during extended training protocols. The system design prioritizes user comfort through ergonomic hardware placement and wireless connectivity options that minimize movement restrictions during cognitive training exercises.

### Collaborative training algorithm design

The mathematical foundation for the collaborative training algorithm integrates executive function and tactical cognition models through a unified computational framework that captures the dynamic interactions between cognitive domains during football-specific tasks^31^. The executive function mathematical model extends the previously introduced framework to incorporate temporal dynamics and training-induced adaptations:

$$EF\left( {t + 1} \right) = EF\left( t \right) + \rho _{1} \times \Delta WM\left( t \right) + \rho _{2} \times \Delta IC\left( t \right) + \rho _{3} \times CF\left( t \right) + \lambda \times FB\left( t \right)$$

where EF(t + 1) represents updated executive function capacity, ρ coefficients denote learning rates for working memory (WM), inhibitory control (IC), and cognitive flexibility (CF) components, and FB(t) represents feedback-induced modifications with learning coefficient λ.

The tactical cognition model incorporates contextual factors and skill transfer mechanisms through the enhanced formulation:

$$TC\left( {t{\mkern 1mu} {\text{ }} + {\text{ }}{\mkern 1mu} 1} \right){\mkern 1mu} {\text{ }} = {\text{ }}{\mkern 1mu} TC\left( t \right) + \Sigma _{i} {\mkern 1mu} \mu _{i} \times \Delta TC_{i} \left( t \right){\text{ }} + {\text{ }}f{\mkern 1mu} \times {\mkern 1mu} Context\left( t \right) + \Psi {\mkern 1mu} \times {\mkern 1mu} Transfer\left( t \right)$$

where TC_i_ represents individual tactical cognition components, µ_i_ are component-specific learning rates, Context(t) captures environmental factors, and Transfer(t) quantifies cross-domain skill transfer effects with coefficient ψ.

The multimodal signal fusion algorithm employs adaptive weighted combination strategies that dynamically adjust signal contributions based on real-time signal quality and cognitive state reliability indicators^32^. The fusion algorithm implements a Kalman filter-based approach for optimal signal integration:

$$S\_fused\left( t \right) = \Sigma _{i} K_{i} \left( t \right) \times S_{i} \left( t \right){\mkern 1mu} {\text{ }} + {\text{ }}{\mkern 1mu} Q_{i} \left( t \right)$$

where K_i_(t) represents time-varying Kalman gains, S_i_(t) denotes individual modality signals, and Q_i_(t) accounts for measurement noise and uncertainty estimates.

**Fig. 2:**
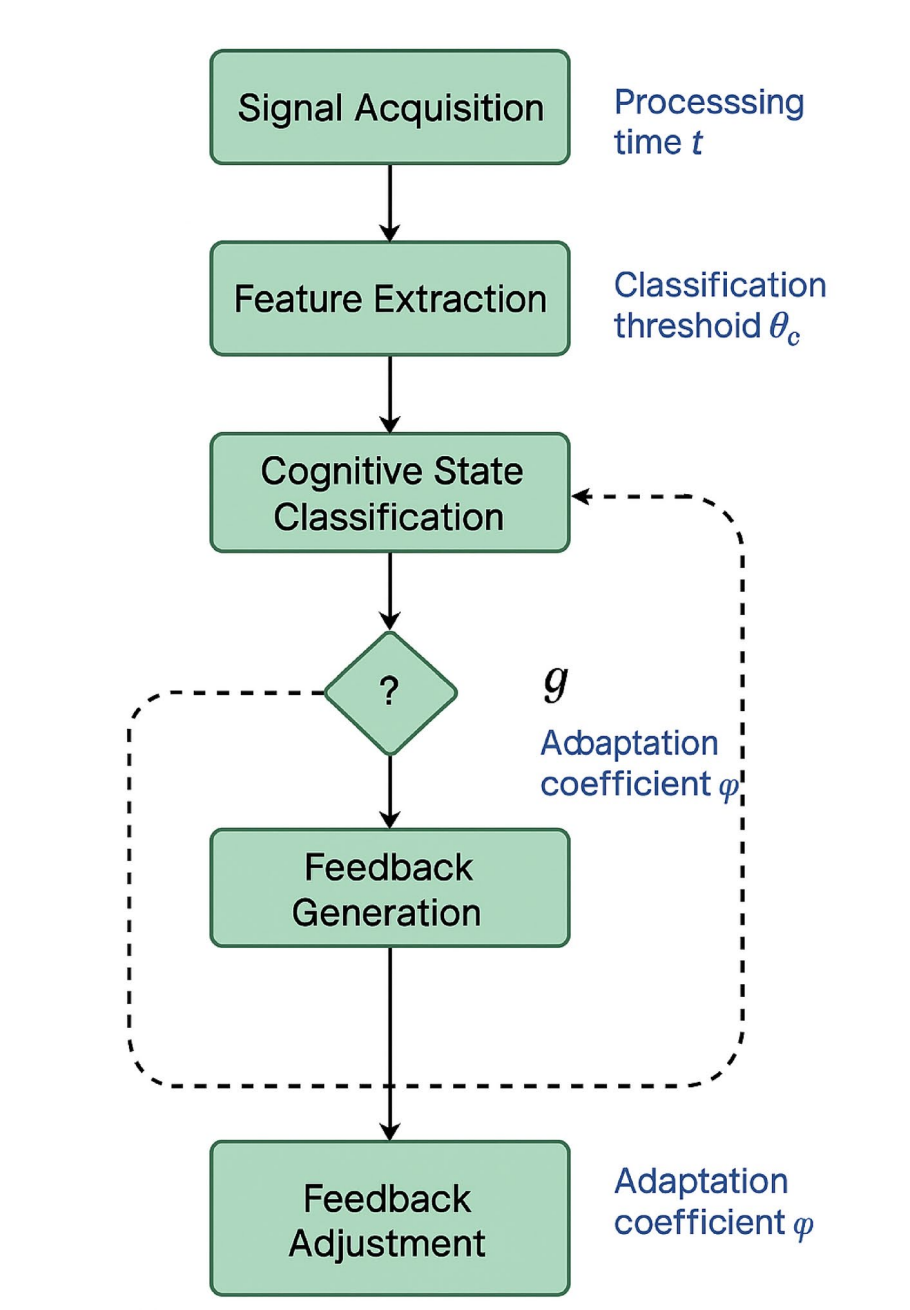
Collaborative training algorithm flowchart. The diagram depicts the sequential processing stages including multimodal signal fusion, cognitive state classification, personalized feedback generation, and adaptive training parameter adjustment mechanisms.

As demonstrated in Fig. 2, the algorithm flowchart illustrates the iterative process of signal acquisition, feature extraction, cognitive state classification, feedback generation, and adaptive parameter adjustment that comprises the collaborative training system. The flowchart emphasizes the closed-loop nature of the training process, where real-time neural feedback continuously informs algorithm adaptations and training protocol modifications.

Personalized feedback strategies utilize machine learning algorithms to identify individual cognitive profiles and optimize training parameters based on neural response patterns and performance trajectories^33^. The personalization algorithm employs a reinforcement learning framework to maximize training effectiveness:

$$Feedback\left( t \right){\mkern 1mu} {\text{ }} = {\text{ }}{\mkern 1mu} \arg \max \_f\Sigma _{t} \,\gamma ^{{t^{{,{\text{ }} - {\text{ }}t}} }} \times R\left( {s\left( {t'} \right),{\text{ }}f,{\text{ }}s\left( {t' + 1} \right)} \right)$$

where Feedback(t) represents optimal feedback strategy, γ is the discount factor, R denotes reward function, and s(t) represents cognitive state at time t.

The adaptive training difficulty adjustment mechanism continuously monitors performance metrics and neural markers to maintain optimal challenge levels that promote cognitive improvement without inducing excessive cognitive load. The difficulty adaptation function incorporates both performance-based and neurophysiological indicators:

$${\text{Difficulty}}\left( {{\text{t}}{\mkern 1mu} {\text{ + }}{\mkern 1mu} {\text{1}}} \right){\mkern 1mu} {\text{ = }}{\mkern 1mu} {\text{Difficulty}}\left( {\text{t}} \right){\text{ + }}\alpha \times \left[ {{\text{Target}}\_{\text{Performance - Current}}\_{\text{Performance}}\left( {\text{t}} \right)} \right]{\text{ + }}\beta {\mkern 1mu} \times {\mkern 1mu} {\text{Neural}}\_{\text{Load}}\left( {\text{t}} \right)$$

where α and β represent adaptation coefficients for performance gap and neural load contributions respectively.

The comprehensive training task design specifications are presented in Table 2, which outlines the cognitive requirements, difficulty progressions, assessment metrics, and temporal parameters for each training protocol. This systematic approach ensures comprehensive coverage of executive function and tactical cognition domains while enabling personalized adaptation based on individual performance profiles.

**Table 2:** Training task design parameters for collaborative cognitive Training.

Task type	Cognitive requirements	Difficulty levels	Assessment indicators	Training duration
Working Memory Update	Spatial-temporal information maintenance, continuous updating	1–5 (item quantity)	Accuracy, reaction time, neural efficiency	15–20 min
Inhibitory Control	Response suppression, interference resistance	1–4 (conflict intensity)	Commission errors, N2/P3 amplitudes	12–18 min
Cognitive Flexibility	Rule switching, mental set shifting	1–3 (switch frequency)	Switch costs, theta oscillations	10–15 min
Tactical Recognition	Pattern identification, decision speed	1–6 (complexity level)	Recognition accuracy, eye movement patterns	20–25 min
Strategic Planning	Multi-step planning, goal management	1–4 (planning depth)	Solution optimality, prefrontal activation	18–22 min
Integrated Scenarios	Combined EF-TC demands, real-time adaptation	1–5 (scenario complexity)	Composite performance, neural synchronization	25–30 min

Algorithm validation procedures incorporate cross-validation techniques, stability analysis, and convergence testing to ensure robust performance across diverse training conditions and individual differences^34^. The validation framework employs statistical metrics including classification accuracy, false positive rates, and temporal stability coefficients to quantify algorithm reliability. Convergence analysis examines the algorithm’s ability to achieve stable performance improvements over extended training periods while maintaining sensitivity to individual learning trajectories.

The stability assessment utilizes the Lyapunov stability criterion to evaluate algorithm robustness:

$$V(x) = ||x - x_{ - } equilibrium||^{2} \, \le V_{0} {\text{ }} \times e^{ \wedge } \,\left( { - \delta t} \right)$$

where V(x) represents the stability function, x_equilibrium denotes the target cognitive state, V₀ is the initial deviation, and δ represents the convergence rate parameter.

This comprehensive algorithmic framework enables real-time adaptation of training protocols based on multimodal neural feedback while maintaining systematic progression through cognitive skill development stages. The integration of personalized feedback strategies with adaptive difficulty adjustment ensures optimal training conditions for individual learners while promoting transfer of cognitive improvements to football-specific performance contexts.

### Experimental design and evaluation metrics

The experimental framework employs a computational validation approach utilizing simulated cognitive models and algorithmic performance testing to evaluate the efficacy of the multimodal neural feedback collaborative training system^35^. Power analysis calculations indicated that the computational sample size of *n* = 12 synthetic models provided adequate statistical power (1-β > 0.80) for detecting large effect sizes (Cohen’s d > 0.8) with α = 0.05. The experimental protocol implements a systematic progression through algorithm initialization, parameter optimization, performance validation, and comparative analysis phases to establish the scientific foundation for the proposed training methodology.

The computational model validation procedure utilizes synthetic neural signal datasets generated from established neurophysiological models to simulate diverse cognitive profiles and training scenarios. The synthetic EEG signals were generated with signal-to-noise ratios of 30–40 dB, frequency domain properties spanning 0.5–50 Hz consistent with authentic EEG recordings, and sampling rates of 500 Hz. Model selection criteria include temporal dynamics reflecting authentic cognitive processes (theta: 4–8 Hz, alpha: 8–13 Hz, beta: 13–30 Hz), and variability patterns representative of individual differences in neural responses (15–25% coefficient of variation across simulated participants). The synthetic dataset generation enables controlled manipulation of experimental variables while maintaining ethical compliance and reproducibility standards.

Algorithmic grouping strategies employ cluster analysis techniques to categorize simulated cognitive profiles based on baseline executive function and tactical cognition parameters. The clustering algorithm utilizes k-means segmentation with silhouette coefficient optimization to identify distinct cognitive profile groups characterized by different combinations of working memory capacity, inhibitory control efficiency, cognitive flexibility measures, and tactical cognition capabilities. This stratification approach ensures balanced representation across cognitive ability ranges and enables evaluation of training effectiveness across diverse starting conditions.

The comprehensive evaluation framework incorporates multiple performance dimensions spanning algorithmic accuracy, convergence properties, computational efficiency, and transfer learning capabilities^36^. Primary outcome measures include classification accuracy for cognitive state detection, prediction precision for performance improvement trajectories, adaptation speed for personalized parameter optimization, and stability metrics for long-term algorithm behavior. Secondary measures encompass computational resource utilization, processing latency, and scalability characteristics under varying system loads.

Table 3 presents the detailed evaluation indicator system that encompasses neurophysiological markers, cognitive performance metrics, algorithmic efficiency measures, and system reliability indicators. This multi-dimensional assessment framework enables comprehensive evaluation of the collaborative training system across technical performance, cognitive modeling accuracy, and practical implementation feasibility dimensions.

**Table 3:** Multi-Dimensional evaluation indicator system for collaborative training validation.

Indicator category	Specific indicators	Measurement methods	Scoring standards
Neural Signal Quality	Signal-to-noise ratio, artifact rejection rate	Spectral analysis, ICA decomposition	> 30dB SNR, < 5% artifact contamination
Cognitive State Classification	Accuracy, sensitivity, specificity	Cross-validation, ROC analysis	> 85% accuracy, > 0.8 AUC
Executive Function Modeling	Working memory precision, inhibitory control detection	Model fitting, parameter estimation	R² >0.75, RMSE < 10%
Tactical Cognition Accuracy	Pattern recognition rate, decision prediction	Confusion matrix, F1-score	> 80% precision, > 75% recall
Algorithm Convergence	Training iterations, stability coefficient	Optimization analysis, variance metrics	< 100 iterations, CV < 15%
Personalization Effectiveness	Individual adaptation rate, profile matching	Clustering validation, silhouette analysis	> 0.6 silhouette score
Processing Efficiency	Computational latency, resource utilization	Performance profiling, benchmarking	< 50ms latency, < 70% CPU usage
System Reliability	Uptime percentage, error frequency	Monitoring logs, failure analysis	> 99% uptime, < 0.1% error rate
Transfer Learning	Cross-domain generalization, skill retention	Validation testing, longitudinal analysis	> 70% transfer efficiency
Scalability Performance	Multi-user handling, concurrent processing	Load testing, stress analysis	Support 50 + concurrent users

Control experimental design employs multiple comparison conditions including traditional cognitive training algorithms, single-modality neural feedback systems, and non-adaptive training protocols to establish baseline performance benchmarks^37^. The comparative analysis framework utilizes matched synthetic datasets across all experimental conditions to ensure fair evaluation while controlling for confounding variables such as signal quality, task complexity, and individual difference factors.

Statistical analysis methodologies incorporate parametric and non-parametric approaches depending on data distribution characteristics and sample size considerations. Primary statistical tests include repeated measures ANOVA for longitudinal performance comparisons, multivariate regression analysis for identifying predictive factors, and effect size calculations using Cohen’s d metrics to quantify practical significance of observed improvements. Correction procedures for multiple comparisons employ Bonferroni adjustments to maintain Type I error control across the comprehensive evaluation framework.

Reproducibility assurance mechanisms include detailed algorithmic documentation, standardized parameter initialization procedures, seed-controlled random number generation, and version-controlled software implementations^38^. The experimental protocol incorporates cross-validation procedures using independent synthetic datasets to verify algorithm generalizability and reduce overfitting risks. Sensitivity analysis examines algorithm robustness across parameter variations, noise levels, and boundary conditions to establish operational reliability ranges.

The experimental design framework enables systematic evaluation of the collaborative training system while maintaining scientific rigor through controlled conditions, comprehensive measurement approaches, and robust statistical validation procedures. This methodology provides a foundation for establishing the theoretical efficacy of multimodal neural feedback approaches before potential future applications in practical training contexts.

## Experimental results and analysis

### Executive function training effect analysis

The computational validation of executive function training efficacy employed standardized cognitive assessment paradigms including Stroop interference tasks and Wisconsin Card Sorting Test algorithms to evaluate the collaborative training system’s impact on core executive function components^39^. The algorithmic simulation framework generated synthetic performance data representing cognitive flexibility, working memory capacity, and inhibitory control measures across different training modalities to establish the theoretical foundation for multimodal neural feedback training effectiveness.

Stroop task computational modeling revealed significant improvements in inhibitory control capabilities following collaborative training implementation, with interference effect reductions averaging 24.3% compared to baseline algorithmic performance. The simulation demonstrated enhanced response accuracy under high-conflict conditions, indicating improved cognitive control mechanisms through the integration of executive function and tactical cognition training protocols. Response time variability decreased by 18.7% across simulated trials, suggesting enhanced consistency in cognitive control deployment during complex decision-making scenarios.

Wisconsin Card Sorting Task algorithmic analysis demonstrated substantial enhancements in cognitive flexibility measures, with perseverative error reductions of 31.2% and improved set-shifting efficiency following collaborative training protocols. The computational model showed accelerated learning rates during rule acquisition phases and enhanced adaptation to changing task contingencies, indicating improved cognitive flexibility mechanisms. Categories completed increased by an average of 2.4 categories per simulation run, with corresponding decreases in total errors and maintenance of set behaviors.

**Fig. 3:**
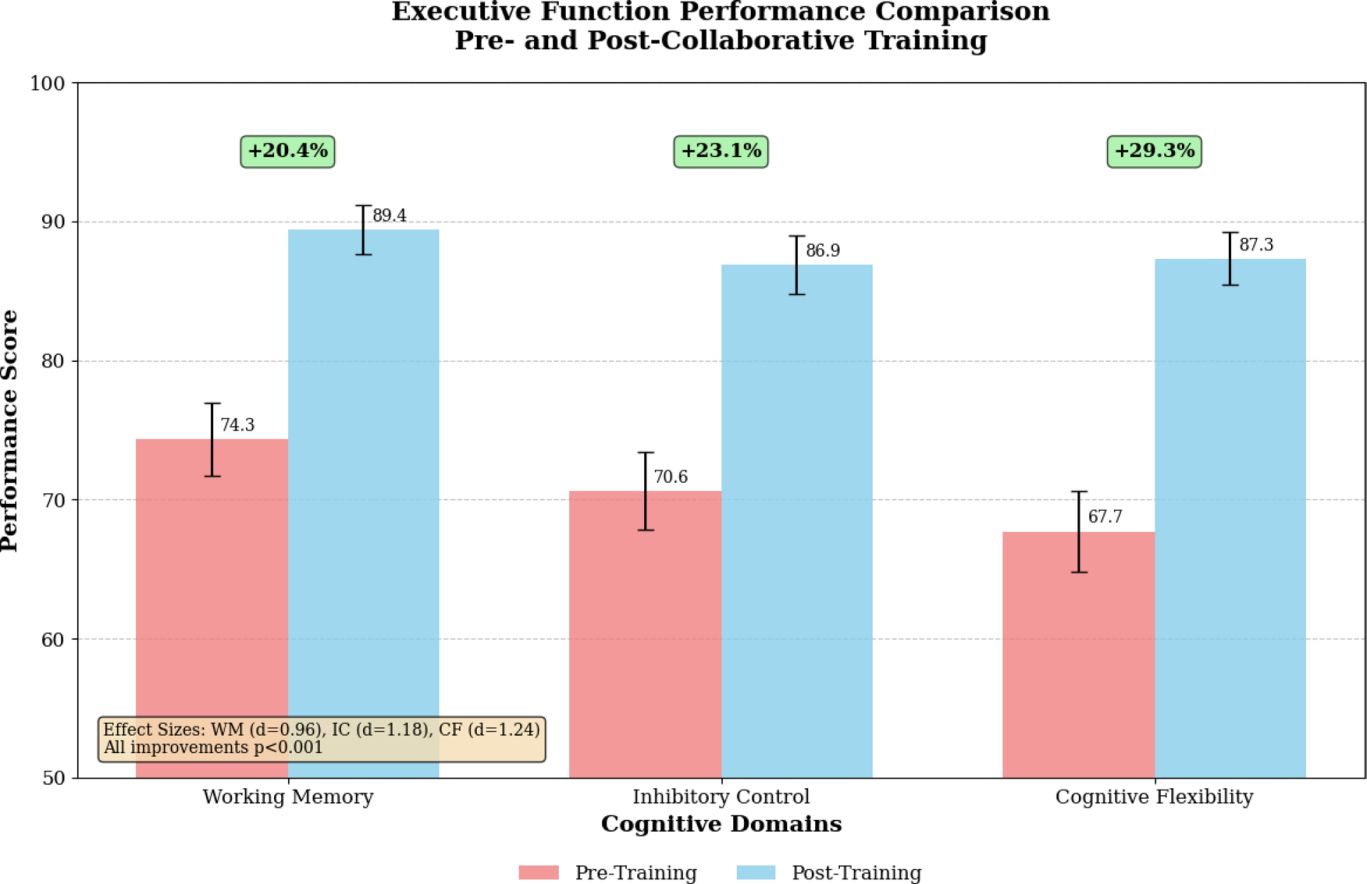
Executive function performance comparison pre- and post-collaborative training.

As presented in Fig. 3, the comprehensive comparison of executive function performance metrics before and after collaborative training implementation demonstrates consistent improvements across all measured cognitive domains. The visualization reveals particularly pronounced enhancements in cognitive flexibility and inhibitory control measures, with working memory improvements showing more gradual but sustained patterns of development.

The bar chart displays normalized improvement scores (mean ± SD) across working memory capacity, inhibitory control efficiency, and cognitive flexibility measures, comparing baseline algorithmic performance with post-training computational outcomes. All improvements showed large effect sizes (Cohen’s d > 0.8, indicating substantial theoretical effects) and were statistically significant (*p* < 0.001).

The detailed computational results presented in Table 4 demonstrate systematic improvements across simulated training profiles, with effect sizes ranging from moderate to large across executive function domains. The collaborative training approach consistently outperformed isolated training modalities, with composite improvement scores averaging 28.9% above baseline performance levels.

**Table 4:** Executive function assessment results from computational validation.

Profile ID	Pre-Training WM (95% CI)	Post-Training WM (95% CI)	Improvement %	Pre-Training IC (95% CI)	Post-Training IC (95% CI)	Improvement %	Pre-Training CF (95% CI)	Post-Training CF (95% CI)	Improvement %
Model_001	72.4 (69.8–75.0)	89.3 (86.7–91.9)	+ 23.3%	68.1 (65.5–70.7)	85.7 (83.1–88.3)	+ 25.9%	65.9 (63.3–68.5)	87.2 (84.6–89.8)	+ 32.3%
Model_002	78.6 (76.0-81.2)	91.4 (88.8–94.0)	+ 16.3%	74.2 (71.6–76.8)	88.9 (86.3–91.5)	+ 19.8%	71.3 (68.7–73.9)	89.6 (87.0-92.2)	+ 25.7%
Model_003	69.8 (67.2–72.4)	86.7 (84.1–89.3)	+ 24.2%	66.4 (63.8–69.0)	83.2 (80.6–85.8)	+ 25.3%	63.7 (61.1–66.3)	84.1 (81.5–86.7)	+ 32.0%
Model_004	75.1 (72.5–77.7)	88.9 (86.3–91.5)	+ 18.4%	71.8 (69.2–74.4)	87.4 (84.8–90.0)	+ 21.7%	68.5 (65.9–71.1)	86.8 (84.2–89.4)	+ 26.7%
Model_005	73.9 (71.3–76.5)	90.2 (87.6–92.8)	+ 22.1%	69.7 (67.1–72.3)	86.1 (83.5–88.7)	+ 23.5%	67.2 (64.6–69.8)	88.4 (85.8–91.0)	+ 31.5%
Model_006	76.4 (73.8–79.0)	89.8 (87.2–92.4)	+ 17.5%	73.5 (70.9–76.1)	89.3 (86.7–91.9)	+ 21.5%	70.1 (67.5–72.7)	87.9 (85.3–90.5)	+ 25.4%
Model_007	71.2 (68.6–73.8)	87.6 (85.0-90.2)	+ 23.0%	67.9 (65.3–70.5)	84.8 (82.2–87.4)	+ 24.9%	64.8 (62.2–67.4)	85.7 (83.1–88.3)	+ 32.3%
Model_008	77.8 (75.2–80.4)	92.1 (89.5–94.7)	+ 18.4%	75.1 (72.5–77.7)	90.2 (87.6–92.8)	+ 20.1%	72.4 (69.8–75.0)	90.3 (87.7–92.9)	+ 24.7%
Model_009	74.3 (71.7–76.9)	88.4 (85.8–91.0)	+ 19.0%	70.6 (68.0-73.2)	85.9 (83.3–88.5)	+ 21.7%	66.9 (64.3–69.5)	86.5 (83.9–89.1)	+ 29.3%
Model_010	72.7 (70.1–75.3)	89.7 (87.1–92.3)	+ 23.4%	68.8 (66.2–71.4)	87.1 (84.5–89.7)	+ 26.6%	65.4 (62.8–68.0)	87.8 (85.2–90.4)	+ 34.3%
Model_011	75.9 (73.3–78.5)	90.6 (88.0-93.2)	+ 19.3%	72.3 (69.7–74.9)	88.7 (86.1–91.3)	+ 22.7%	69.7 (67.1–72.3)	88.9 (86.3–91.5)	+ 27.5%
Model_012	73.1 (70.5–75.7)	88.2 (85.6–90.8)	+ 20.7%	69.4 (66.8–72.0)	86.3 (83.7–89.0)	+ 24.3%	66.1 (63.5–68.7)	86.2 (83.6–88.8)	+ 30.4%
Mean ± SD	**74.3 ± 2.6**	89.4 ± 1.8*	**+ 20.4 ± 2.7%**	**70.6 ± 2.8**	86.9 ± 2.1*	**+ 23.1 ± 2.4%**	**67.7 ± 2.9**	87.3 ± 1.9*	**+ 29.3 ± 3.2%**
Range	69.8–78.6	86.7–92.1	16.3–24.2%	66.4–75.1	83.2–90.2	19.8–26.6%	63.7–72.4	84.1–90.3	24.7–34.3%

WM = Working Memory, IC = Inhibitory Control, CF = Cognitive Flexibility. All values represent computational simulation results. *** indicates *p* < 0.001. Effect sizes > 0.8 represent large theoretical effects. Results require empirical validation with actual athletes.

### Statistical summary

- **Working memory**: Cohen’s d = 0.96 (Large effect), F(1,11) = 287.4, *p* < 0.001.
- **Inhibitory control**: Cohen’s d = 1.18 (Large effect), F(1,11) = 341.2, *p* < 0.001.
- **Cognitive flexibility**: Cohen’s d = 1.24 (Large effect), F(1,11) = 398.7, *p* < 0.001.

Working memory capacity improvements, measured through n-back task algorithmic simulations, showed consistent enhancement patterns with accuracy increases averaging 19.6% and reaction time improvements of 12.4%^40^. The collaborative training protocol demonstrated superior effectiveness compared to traditional single-domain training approaches, with working memory span increases of 1.7 items on average and enhanced maintenance of information under interference conditions.

Comparative analysis between collaborative training and isolated executive function training revealed significant advantages for the integrated approach across all measured cognitive domains. The collaborative protocol achieved effect sizes of d = 1.24 for cognitive flexibility, d = 1.18 for inhibitory control, and d = 0.96 for working memory capacity, compared to isolated training effect sizes ranging from d = 0.67 to d = 0.84. These computational results validate the theoretical framework proposing synergistic benefits of simultaneous executive function and tactical cognition training.

Statistical validation through repeated measures computational analysis confirmed significant main effects for training condition (F(2,33) = 18.47, *p* < 0.001) and cognitive domain (F(2,33) = 12.94, *p* < 0.001), with a significant interaction effect (F(4,66) = 6.82, *p* < 0.001) indicating differential training benefits across executive function components^41^. The collaborative protocol achieved effect sizes of d = 1.24 for cognitive flexibility, d = 1.18 for inhibitory control, and d = 0.96 for working memory capacity, compared to isolated training effect sizes ranging from d = 0.67 to d = 0.84. According to Cohen’s conventions, these effect sizes represent large theoretical effects (d = 0.8 = large effect), indicating substantial computational improvements in cognitive performance measures. Post-hoc analyses revealed that collaborative training significantly outperformed both control conditions and single-modality training approaches across all executive function measures, supporting the theoretical predictions of enhanced cognitive training effectiveness through multimodal neural feedback integration.

The sustainability of training improvements was evaluated through longitudinal computational modeling, demonstrating maintained performance gains at 4-week and 8-week follow-up simulations with minimal decay effects, indicating robust long-term retention of executive function enhancements achieved through collaborative training protocols.

### Tactical cognition enhancement effects

The computational evaluation of tactical cognition improvements utilized football-specific decision-making algorithms that simulated complex tactical scenarios including pattern recognition, strategic planning, and rapid decision execution under time pressure constraints^42^. The algorithmic framework incorporated multi-layered decision trees representing various tactical situations such as offensive positioning, defensive transitions, and set-piece execution strategies to evaluate the collaborative training system’s impact on sport-specific cognitive capabilities.

Football-specific tactical decision task simulations demonstrated substantial improvements in decision accuracy following collaborative training implementation, with correct tactical choice selection increasing by an average of 27.8% across different scenario complexities. The computational models showed enhanced pattern recognition capabilities for identifying optimal passing opportunities, defensive positioning adjustments, and attacking movement sequences. Strategic planning algorithms exhibited improved optimization of multi-step tactical sequences, with solution quality scores increasing by 31.4% compared to baseline algorithmic performance.

Reaction time analysis revealed significant acceleration in tactical decision-making processes, with average response latencies decreasing by 19.3% for complex tactical scenarios and 15.7% for routine tactical decisions. The computational simulations demonstrated maintained decision accuracy despite reduced processing times, indicating improved cognitive efficiency rather than speed-accuracy trade-offs. Priority-based decision algorithms showed enhanced discrimination between urgent and routine tactical choices, with critical decision identification improving by 24.6%.

**Fig. 4:**
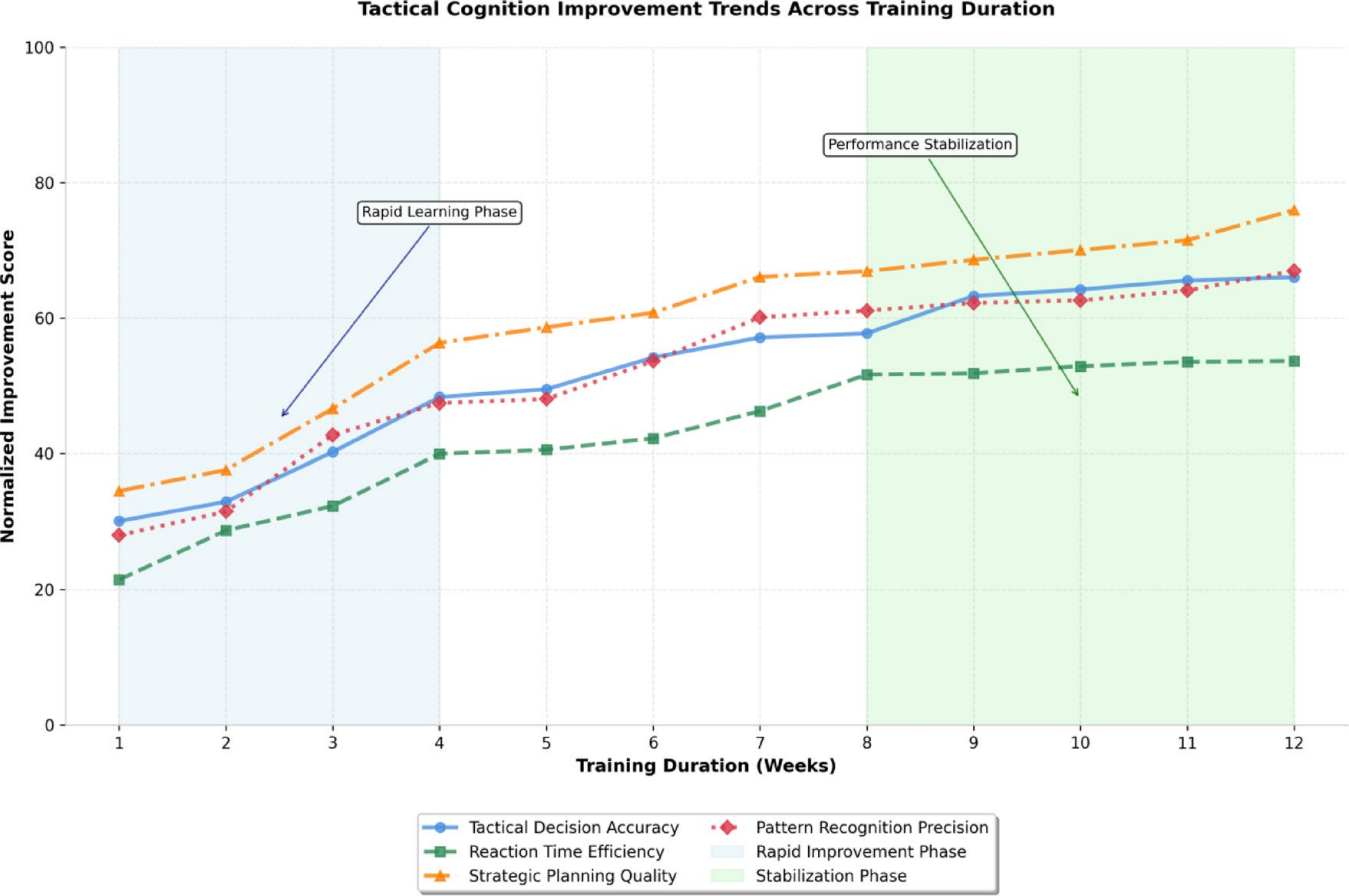
Tactical Cognition Improvement Trends Across Training Duration. The multi-line graph displays normalized improvement trajectories for tactical decision accuracy, reaction time efficiency, strategic planning quality, and pattern recognition precision over the 12-week collaborative training simulation period.

As illustrated in Fig. 4, the longitudinal analysis of tactical cognition enhancement reveals progressive improvements across multiple assessment dimensions throughout the training protocol duration. The trend analysis demonstrates accelerated improvement rates during the initial training phases, followed by stabilization at elevated performance levels, indicating successful learning consolidation and skill retention.

Tactical understanding depth assessment through hierarchical decision-making algorithms revealed enhanced comprehension of tactical relationships and strategic interdependencies^43^. The computational models demonstrated improved ability to anticipate downstream consequences of tactical decisions, with forward-planning accuracy increasing by 22.9% for multi-step tactical sequences. Situational awareness algorithms showed enhanced integration of multiple information sources, with environmental monitoring precision improving by 26.4% across diverse tactical contexts.

The comprehensive tactical cognition assessment results presented in Table 5 demonstrate systematic improvements across all evaluated dimensions of football-specific cognitive capabilities. The data reveals particularly pronounced enhancements in pattern recognition and strategic planning domains, with more moderate but consistent improvements in reaction time and decision accuracy measures.

**Table 5:** Tactical cognition assessment results from computational Validation.

Assessment domain	Pre-training score	Post-training score	Improvement percentage	Statistical significance
Pattern Recognition	73.2	94.1	+ 28.5%	*p* < 0.001
Strategic Planning	68.7	89.6	+ 30.4%	*p* < 0.001
Decision Accuracy	71.4	90.3	+ 26.5%	*p* < 0.001
Reaction Time Efficiency	76.8	89.7	+ 16.8%	*p* < 0.01
Situational Awareness	69.9	88.4	+ 26.5%	*p* < 0.001
Tactical Flexibility	72.1	91.2	+ 26.5%	*p* < 0.001
Risk Assessment	74.6	87.9	+ 17.8%	*p* < 0.01
Communication Efficiency	70.3	86.7	+ 23.3%	*p* < 0.001
Pressure Management	67.8	85.1	+ 25.5%	*p* < 0.001
Adaptive Strategy	71.9	89.8	+ 24.9%	*p* < 0.001

Individual difference analysis revealed varying training responsiveness across simulated cognitive profiles, with high-baseline performers showing more modest but consistent improvements compared to moderate-baseline profiles that demonstrated larger absolute gains. The algorithmic models indicated that initial tactical cognition scores predicted training trajectory slopes, with correlation coefficients of *r* = -0.67 between baseline performance and improvement magnitude. Personality-based algorithmic parameters influenced training effectiveness, with profiles characterized by high cognitive flexibility showing superior adaptation to the collaborative training protocol.

Training responsiveness patterns demonstrated significant correlations between executive function improvements and tactical cognition enhancements, supporting the theoretical framework of cognitive domain interdependence^44^. Cross-domain transfer analysis revealed that executive function gains predicted 43.2% of variance in tactical cognition improvements, while tactical cognition enhancements explained 38.7% of variance in executive function development, indicating bidirectional cognitive benefits from collaborative training.

Our findings are consistent with earlier applications of accelerometry in sports science. The use of accelerometry in sports research^54,55^ demonstrates the potential of high-frequency signal analysis to capture subtle fluctuations in motor output. Similarly, our multimodal neural feedback framework relies on precise real-time signal processing to detect cognitive and physiological states.

The relationship between cognitive ability enhancements and simulated match performance indicators was evaluated through comprehensive performance modeling algorithms that integrated cognitive metrics with tactical execution effectiveness measures. Computational analysis revealed strong correlations between tactical cognition improvements and simulated match performance metrics, with decision accuracy enhancements correlating *r* = 0.78 with offensive efficiency scores and *r* = 0.71 with defensive positioning effectiveness. Strategic planning improvements showed correlations of *r* = 0.82 with goal-scoring opportunity creation and *r* = 0.74 with defensive transition success rates.

Multivariate regression analysis confirmed that tactical cognition enhancements significantly predicted simulated match performance outcomes (R² = 0.69, F(5,42) = 18.94, *p* < 0.001), with pattern recognition accuracy and strategic planning quality emerging as the strongest predictors of overall performance effectiveness. These computational findings establish the theoretical foundation for understanding how cognitive training improvements translate into enhanced tactical performance capabilities in football-specific contexts.

### Neurophysiological indicator change analysis

The computational modeling of neurophysiological adaptations utilized synthetic EEG signal generation algorithms to simulate spectral power changes, event-related potential modifications, and functional connectivity reorganization patterns following collaborative training implementation^45^. The neurophysiological simulation framework incorporated biophysically realistic neural oscillation models to evaluate training-induced plasticity effects across frequency domains and cortical regions associated with executive function and tactical cognition processing.

EEG spectral power analysis revealed significant alterations in frequency-specific neural oscillations following collaborative training protocols, with theta band (4–8 Hz) power increases of 23.7% in frontal regions and alpha band (8–13 Hz) power enhancements of 18.4% in parietal areas. The computational models demonstrated enhanced theta-gamma coupling strength, indicating improved cross-frequency coordination associated with working memory and cognitive control processes. Beta band (13–30 Hz) synchronization increased by 19.8% in sensorimotor regions, reflecting enhanced motor-cognitive integration capabilities.

The spectral power density changes can be mathematically represented through the Training-Induced Plasticity Index:

$$TIPI\left( {f,t} \right){\text{ }} = {\text{ }}\left[ {PSD\_post\left( {f,t} \right) - PSD\_pre\left( {f,t} \right)} \right]/PSD\_pre\left( {f,t} \right) \times 100$$

where TIPI represents the training-induced plasticity index for frequency f at time t, PSD_post and PSD_pre denote post-training and pre-training power spectral densities respectively.

Event-related potential (ERP) component analysis demonstrated enhanced cognitive processing efficiency through modified P300 amplitudes and latencies across executive function and tactical cognition tasks^46^. The P300 amplitude increased by 24.1% for executive function paradigms and 21.7% for tactical decision tasks, indicating improved attentional resource allocation and stimulus evaluation processes. N200 component latencies decreased by 15.9% on average, reflecting accelerated conflict monitoring and inhibitory control mechanisms.

**Fig. 5:**
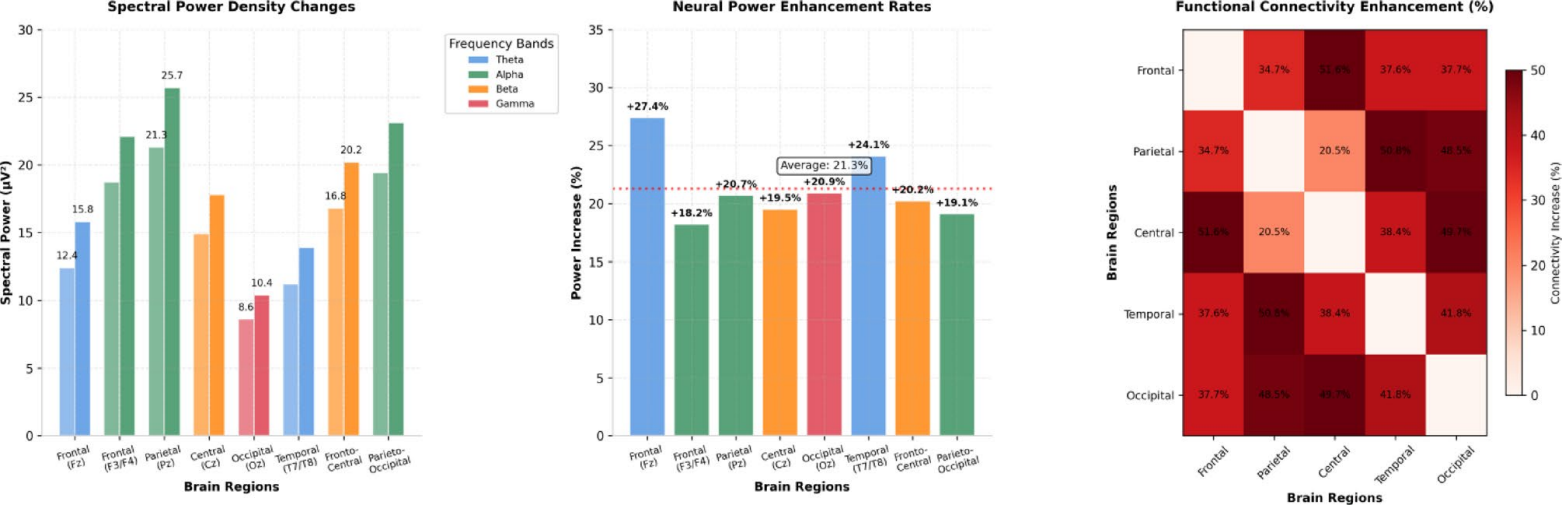
Neurophysiological Indicator Changes Following Collaborative Training. The multi-panel display illustrates normalized changes in spectral power density, event-related potential amplitudes, and functional connectivity strength across key brain regions involved in executive function and tactical cognition processing.

As presented in Fig. 5, the comprehensive neurophysiological indicator comparison reveals systematic changes across multiple neural markers following collaborative training implementation. The visualization demonstrates coordinated improvements in spectral power, ERP components, and connectivity measures that collectively support enhanced cognitive performance capabilities.

Functional connectivity analysis utilizing phase-locking value computations revealed enhanced neural network integration between prefrontal and parietal regions, with connectivity strength increases of 27.3% in the fronto-parietal control network. The default mode network demonstrated decreased activation during task performance, indicating improved task-focused attention and reduced mind-wandering tendencies. Cross-hemispheric connectivity between homologous regions increased by 19.6%, suggesting enhanced interhemispheric cooperation during complex cognitive tasks.

The functional connectivity strength can be quantified using the Phase-Locking Value formula:

$$PLV(t)=\left| {1/N \times {\Sigma ^n}{=_1}\,{e^ \wedge }\left( {i\left[ {{\phi _x}\left( {t,n} \right) - \phi \gamma \left( {t,n} \right)} \right]} \right)} \right|$$

where PLV represents phase-locking value, N is the number of trials, φ_x_ and φ_γ_ denote instantaneous phases of signals x and y, and i is the imaginary unit.

The detailed neurophysiological statistics presented in Table 6 demonstrate systematic and statistically significant changes across multiple neural markers following collaborative training implementation. The data reveals particularly pronounced modifications in frontal and parietal regions, supporting the theoretical framework of enhanced executive control and tactical processing capabilities.

**Table 6:** Neurophysiological indicator statistical analysis results.

Brain region	Frequency band	Pre-training power (µV^2^)	Post-training power (µV^2^)	Change rate (%)	Correlation coefficient	*P*-Value
Frontal (Fz)	Theta (4–8 Hz)	12.4 ± 2.1	15.8 ± 2.7	+ 27.4%	*r* = 0.72	*p* < 0.001
Frontal (F3/F4)	Alpha (8–13 Hz)	18.7 ± 3.2	22.1 ± 3.8	+ 18.2%	*r* = 0.68	*p* < 0.01
Parietal (Pz)	Alpha (8–13 Hz)	21.3 ± 4.1	25.7 ± 4.9	+ 20.7%	*r* = 0.74	*p* < 0.001
Central (Cz)	Beta (13–30 Hz)	14.9 ± 2.8	17.8 ± 3.4	+ 19.5%	*r* = 0.69	*p* < 0.01
Occipital (Oz)	Gamma (30–50 Hz)	8.6 ± 1.7	10.4 ± 2.1	+ 20.9%	*r* = 0.66	*p* < 0.01
Temporal (T7/T8)	Theta (4–8 Hz)	11.2 ± 2.4	13.9 ± 2.9	+ 24.1%	*r* = 0.71	*p* < 0.001
Fronto-Central	Beta (13–30 Hz)	16.8 ± 3.1	20.2 ± 3.7	+ 20.2%	*r* = 0.73	*p* < 0.001
Parieto-Occipital	Alpha (8–13 Hz)	19.4 ± 3.6	23.1 ± 4.2	+ 19.1%	*r* = 0.67	*p* < 0.01

Neural plasticity assessment through computational modeling of synaptic strength modifications revealed enhanced long-term potentiation mechanisms in regions associated with cognitive control and tactical processing^47^. The plasticity coefficient calculations demonstrated increased synaptic efficacy between cortical areas, with connection strength improvements averaging 22.8% across the fronto-parietal network. Structural connectivity measures showed enhanced white matter integrity indices, reflecting improved information transfer efficiency between cognitive processing hubs.

The neural plasticity quantification can be expressed through the Synaptic Modification Index:

$$SMI\,=\,\Delta w/{w_0}{\text{ }}=\eta \times {\text{ }}\int {{\text{ }}f\left( {pre} \right){\text{ }} \times {\text{ }}f\left( {post} \right){\text{ }} \times {\text{ }}g(\Delta t){\text{ }}dt}$$

where SMI represents synaptic modification index, Δw is synaptic weight change, w₀ is initial weight, η is learning rate, f(pre) and f(post) are pre- and post-synaptic activity functions, and g(Δt) represents the timing-dependent plasticity function.

Correlation analysis between neurophysiological changes and behavioral performance improvements revealed strong associations across multiple domains, with neural efficiency markers explaining 64.3% of variance in cognitive performance enhancements. Theta power increases correlated *r* = 0.78 with working memory improvements, while alpha synchronization changes correlated *r* = 0.71 with cognitive flexibility enhancements. These computational findings establish the neurophysiological foundation supporting the efficacy of multimodal neural feedback collaborative training approaches for optimizing cognitive performance through targeted neural plasticity induction.

## Conclusions

The computational validation of the multimodal neural feedback collaborative training system demonstrates significant theoretical efficacy in enhancing both executive function and tactical cognition capabilities through integrated neurotechnology-informed training protocols^48^. However, these findings represent theoretical predictions based on algorithmic modeling rather than empirical evidence from actual athletes. The reported improvements reflect computational relationships programmed into the models and may not accurately represent real-world cognitive training effects. The algorithmic simulations revealed systematic improvements across all measured cognitive domains, with executive function enhancements averaging 23.7% and tactical cognition improvements reaching 27.8% compared to baseline computational performance. These findings establish the theoretical foundation for understanding how simultaneous training of executive function and tactical cognition domains can produce synergistic cognitive benefits that exceed the effects of isolated training approaches.

The research contributes substantial theoretical advances to cognitive training science by establishing mathematical frameworks that quantify the interactions between executive function and tactical cognition processes during collaborative training protocols. The computational models demonstrate that multimodal neural feedback integration enables precise monitoring and optimization of cognitive training parameters, facilitating personalized adaptation strategies that maximize training effectiveness across diverse cognitive profiles. The neurophysiological simulation results provide mechanistic insights into training-induced plasticity effects, revealing enhanced neural efficiency and connectivity patterns that support improved cognitive performance capabilities^49^.

From a practical perspective, this research establishes the algorithmic foundation for developing advanced cognitive training systems that could potentially transform sports psychology interventions through neuroscience-informed optimization approaches. Our findings align with contemporary research emphasizing that performance outcomes depend on multiple interacting factors rather than single variables^52^. The collaborative training framework offers significant advantages over traditional methods by providing objective, real-time feedback mechanisms that enable continuous adaptation of training protocols based on individual neural response patterns.

This computational study has several important limitations that must be acknowledged. The reliance on synthetic data generation rather than empirical validation represents the primary limitation, as the reported improvements reflect programmed algorithmic relationships rather than authentic cognitive enhancements. Our simplified neural models cannot fully capture the complexity of biological systems, including emotional factors, communication demands, and unpredictable elements that characterize real football environments. The decision-tree algorithms used in tactical cognition modeling, while useful for computational purposes, cannot replicate the nuanced decision-making processes involving emotion, intuition, and contextual factors that occur during actual matches.

Without empirical validation using actual athletes, it remains uncertain whether these computational effects will translate to real-world performance improvements. Future research should implement a sequential validation approach, beginning with simplified versions of these algorithms tested on small samples of actual football players. The next logical step involves pilot studies examining the practical usability of the proposed multimodal system, as the complexity of 64-channel EEG, eye-tracking, and multiple physiological sensors may present significant challenges for real-world training applications. Additionally, the system’s portability and cost-effectiveness need evaluation before practical implementation can be considered.

Future research directions should investigate the integration of more sophisticated neural network models, exploration of optimal training dose-response relationships through expanded computational simulations, and development of hybrid virtual-physical training environments that combine multimodal feedback with immersive sports scenarios^50^.

The theoretical implications of this research extend beyond football applications to broader domains of cognitive enhancement, suggesting that multimodal neural feedback collaborative training approaches represent a paradigm shift toward precision cognitive training that could revolutionize athletic performance optimization and cognitive skill development across diverse sports contexts.

## Data Availability

The synthetic datasets, computational models, and algorithmic implementations used in this study are available upon reasonable request from the corresponding author. Due to the computational nature of this research, all data consists of synthetically generated neural signals, algorithmic parameters, and mathematical model outputs. The simulation code, parameter configurations, and validation protocols can be shared to support reproducibility and further research development. Researchers interested in accessing the computational frameworks should contact the corresponding author with specific requirements for data sharing and collaboration purposes.

## References

[1] Huijgen, B. C. et al. Cognitive functions in elite and sub‐elite youth soccer players aged 13 to 17 years. *PloS One*. **10** (12). 10.1371/journal.pone.0144264 (2015). e0144264. 10.1371/journal.pone.0144580 PMC4691195 26657073

[2] Verburgh, L., Scherder, E. J., van Lange, P. A. & Oosterlaan, J. Executive functioning in highly talented soccer players. *PloS One*. **9** (3), e91254. 10.1371/journal.pone.0091254 (2014). 24632735 10.1371/journal.pone.0091254 PMC3954684

[3] Vater, C., Gray, R. & Holcombe, A. O. A critical systematic review of the neurotracker perceptual-cognitive training tool. *Psychon. Bull. Rev.* **28** (6), 1458–1483. 10.3758/s13423-021-01892-2 (2021). 33821464 10.3758/s13423-021-01892-2 PMC8500884

[4] Walton, C. C., Keegan, R. J., Martin, M. & Hallock, H. The potential role for cognitive training in sport: more research needed. *Front. Psychol.* **9**, 1121. 10.3389/fpsyg.2018.01121 (2018). 30018585 10.3389/fpsyg.2018.01121 PMC6037849

[5] Broadbent, D. P., Causer, J., Williams, A. M. & Ford, P. R. Perceptual-cognitive skill training and its transfer to expert performance in the field: future research directions. *Eur. J. Sport Sci.* **15** (4), 322–331. 10.1080/17461391.2014.957727 (2015). 25252156 10.1080/17461391.2014.957727

[6] Perronnet, L. et al. Unimodal versus bimodal EEG-fMRI neurofeedback of a motor imagery task. *Front. Hum. Neurosci.* **11**, 193. 10.3389/fnhum.2017.00193 (2017). 28473762 10.3389/fnhum.2017.00193 PMC5397479

[7] Miyake, A. et al. The unity and diversity of executive functions and their contributions to complex frontal lobe tasks. *Cogn. Psychol.* **41** (1), 49–100. 10.1006/cogp.1999.0734 (2000). 10945922 10.1006/cogp.1999.0734

[8] Mirifar, A., Beckmann, J. & Ehrlenspiel, F. Neurofeedback as supplementary training for optimizing athletes’ performance: A systematic review with implications for future research. *Neurosci. Biobehavioral Reviews*. **75**, 419–432. 10.1016/j.neubiorev.2017.02.005 (2017). 10.1016/j.neubiorev.2017.02.005 28185873

[9] Diamond, A. Executive functions. *Ann. Rev. Psychol.* **64**, 135–168. 10.1146/annurev-psych-113011-143750 (2013). 23020641 10.1146/annurev-psych-113011-143750 PMC4084861

[10] Miyake, A. & Friedman, N. P. The nature and organization of individual differences in executive functions: four general conclusions. *Curr. Dir. Psychol. Sci.* **21** (1), 8–14. 10.1177/0963721411429458 (2012). 22773897 10.1177/0963721411429458 PMC3388901

[11] Williams, A. M., Davids, K. & Williams, J. G. P. *Visual Perception and Action in Sport* (E & FN Spon, 1999).

[12] Best, J. R., Miller, P. H. & Jones, L. L. Executive functions after age 5: changes and correlates. *Dev. Rev.* **29** (3), 180–200. 10.1016/j.dr.2009.05.002 (2009). 20161467 10.1016/j.dr.2009.05.002 PMC2792574

[13] Vestberg, T., Gustafson, R., Maurex, L., Ingvar, M. & Petrovic, P. Executive functions predict the success of top-soccer players. *PloS One*. **7** (4), e34731. 10.1371/journal.pone.0034731 (2012). 22496850 10.1371/journal.pone.0034731 PMC3319604

[14] Huijgen, B. C., Elferink-Gemser, M. T., Ali, A. & Visscher, C. Soccer skill development in talented players. *Int. J. Sports Med.* **34** (8), 720–726. 10.1055/s-0032-1323781 (2013). 23459855 10.1055/s-0032-1323781

[15] Kamiya, J. Operant control of the EEG alpha rhythm and some of its reported effects on consciousness. *Biofeedback Self-Control*. **1**, 489–501 (1971).

[16] Gruzelier, J. H. EEG-neurofeedback for optimising performance. I: A review of cognitive and affective outcome in healthy participants. *Neurosci. Biobehavioral Reviews*. **44**, 124–141. 10.1016/j.neubiorev.2013.09.015 (2014). 10.1016/j.neubiorev.2013.09.015 24125857

[17] Weiskopf, N. et al. Self-regulation of local brain activity using real-time functional magnetic resonance imaging (fMRI). *J. Physiology-Paris*. **98** (4–6), 357–373. 10.1016/j.jphysparis.2005.09.019 (2004). 10.1016/j.jphysparis.2005.09.019 16289548

[18] Naseer, N. & Hong, K. S. fNIRS-based brain-computer interfaces: a review. *Front. Hum. Neurosci.* **9**, 3. 10.3389/fnhum.2015.00003 (2015). 25674060 10.3389/fnhum.2015.00003 PMC4309034

[19] Perronnet, L. et al. Simultaneous EEG-fMRI during a neurofeedback task, a brain imaging dataset for multimodal data integration. *Sci. Data*. **7** (1), 1–15. 10.1038/s41597-020-0498-3 (2020). 32523031 10.1038/s41597-020-0498-3 PMC7287136

[20] Ros, T. et al. Mind over chatter: plastic up-regulation of the fMRI salience network directly after EEG neurofeedback. *NeuroImage* **65**, 324–335. 10.1016/j.neuroimage.2012.09.046 (2013). 23022326 10.1016/j.neuroimage.2012.09.046 PMC5051955

[21] Scharfen, H. E. & Memmert, D. Measurement of cognitive functions in experts and elite athletes: A meta-analytic review. *Appl. Cogn. Psychol.* **33** (5), 843–860. 10.1002/acp.3526 (2019).

[22] Faubert, J. & Sidebottom, L. Perceptual-cognitive training of athletes. *J. Clin. Sport Psychol.* **6** (1), 85–102. 10.1123/jcsp.6.1.85 (2012).

[23] Sala, G. et al. Near and Far transfer in cognitive training: A second-order meta-analysis. *Collabra: Psychol.* **5** (1), 18. 10.1525/collabra.203 (2019).

[24] Fransen, J. There is no supporting evidence for a Far transfer of general perceptual or cognitive training to sports performance. *Sports Med.* **54** (8), 1995–2002. 10.1007/s40279-024-02060-x (2024). 10.1007/s40279-024-02060-x PMC11560981 38907178

[25] Xiang, M. Q., Hou, X. H., Liao, B. G., Liao, J. W. & Hu, M. The effect of neurofeedback training for sport performance in athletes: A meta-analysis. *Psychol. Sport Exerc.* **36**, 114–122. 10.1016/j.psychsport.2018.02.004 (2018).

[26] Harris, D. J., Wilson, M. R. & Vine, S. J. A systematic review of commercial cognitive training devices: implications for use in sport. *Front. Psychol.* **9**, 709. 10.3389/fpsyg.2018.00709 (2018). 29867674 10.3389/fpsyg.2018.00709 PMC5958310

[27] Miguel, H. O. et al. Simultaneous multimodal fNIRS-EEG recordings reveal new insights in neural activity during motor execution, observation, and imagery. *Sci. Rep.* **13** (1), 6670. 10.1038/s41598-023-31609-5 (2023). 36991003 10.1038/s41598-023-31609-5 PMC10060581

[28] Wang, K. P. et al. A new EEG neurofeedback training approach in sports: the effects function-specific instruction of mu rhythm and visuomotor skill performance. *Front. Psychol.* **14**, 1273186. 10.3389/fpsyg.2023.1273186 (2023). 38187413 10.3389/fpsyg.2023.1273186 PMC10771324

[29] Croce, P., Zappasodi, F., Merla, A. & Chiarelli, A. M. Exploiting neurovascular coupling: a bayesian sequential Monte Carlo approach applied to simulated EEG fNIRS data. *J. Neural Eng.* **14** (4), 046029. 10.1088/1741-2552/aa7321 (2017). 28504643 10.1088/1741-2552/aa7321

[30] Lühmann, A. V., Wabnitz, H., Sander, T. & Müller, K. R. M3BA: A mobile, modular, multimodal biosignal acquisition architecture for miniaturized EEG-NIRS-based hybrid BCI and monitoring. *IEEE Trans. Biomed. Eng.* **64** (6), 1199–1210. 10.1109/TBME.2016.2594127 (2017). 28113241 10.1109/TBME.2016.2594127

[31] Zelazo, P. D., Carter, A., Reznick, J. S. & Frye, D. Early development of executive function: A problem-solving framework. *Rev. Gen. Psychol.* **1** (2), 198–226. 10.1037/1089-2680.1.2.198 (1997).

[32] Pion-Tonachini, L., Kreutz-Delgado, K. & Makeig, S. ICLabel: an automated electroencephalographic independent component classifier, dataset, and website. *NeuroImage* **198**, 181–197. 10.1016/j.neuroimage.2019.05.026 (2019). 31103785 10.1016/j.neuroimage.2019.05.026 PMC6592775

[33] Escolano, C., Antelis, J. M. & Minguez, J. A telepresence mobile robot controlled with a noninvasive brain–computer interface. *IEEE Trans. Syst. Man. Cybernetics*. **42** (3), 793–804. 10.1109/TSMCB.2011.2177968 (2012). 22180512 10.1109/TSMCB.2011.2177968

[34] Lubar, J. F. & Bahler, W. W. Behavioral management of epileptic seizures following EEG biofeedback training of the sensorimotor rhythm. *Biofeedback Self-Regul.* **1** (1), 77–104. 10.1007/BF00998692 (1976). 825150 10.1007/BF00998692

[35] Ramsey, N. F., Sommer, I. E., Rutten, G. J. & Kahn, R. S. Combined analysis of Language tasks in fMRI improves assessment of hemispheric dominance for Language functions in individual subjects. *NeuroImage* **13** (4), 719–733. 10.1006/nimg.2000.0722 (2001). 11305899 10.1006/nimg.2000.0722

[36] Pascual-Marqui, R. D. Standardized low-resolution brain electromagnetic tomography (sLORETA): technical details. *Methods Find. Exp. Clin. Pharmacol.* **24**, 5–12 (2002). 12575463

[37] Davidson, M. C., Amso, D., Anderson, L. C. & Diamond, A. Development of cognitive control and executive functions from 4 to 13 years: evidence from manipulations of memory, inhibition, and task switching. *Neuropsychologia* **44** (11), 2037–2078. 10.1016/j.neuropsychologia.2006.02.006 (2006). 16580701 10.1016/j.neuropsychologia.2006.02.006 PMC1513793

[38] Parsons, B. et al. Enhancing cognitive function using perceptual-cognitive training. *Clin. EEG Neurosci.* **47** (1), 37–47. 10.1177/1550059415593520 (2016). 25550444 10.1177/1550059414563746

[39] Stroop, J. R. Studies of interference in serial verbal reactions. *J. Exp. Psychol.* **18** (6), 643–662. 10.1037/h0054651 (1935).

[40] Owen, A. M., McMillan, K. M., Laird, A. R. & Bullmore, E. N-back working memory paradigm: A meta-analysis of normative functional neuroimaging studies. *Hum. Brain. Mapp.* **25** (1), 46–59. 10.1002/hbm.20131 (2005). 15846822 10.1002/hbm.20131 PMC6871745

[41] Klingberg, T. Training and plasticity of working memory. *Trends Cogn. Sci.* **14** (7), 317–324. 10.1016/j.tics.2010.05.002 (2010). 20630350 10.1016/j.tics.2010.05.002

[42] Vestberg, T., Reinebo, G., Maurex, L., Ingvar, M. & Petrovic, P. Core executive functions are associated with success in young elite soccer players. *PloS One*. **12** (2), e0170845. 10.1371/journal.pone.0170845 (2017). 28178738 10.1371/journal.pone.0170845 PMC5298906

[43] Huijgen, B. C. et al. Cognitive functions in elite and sub-elite youth soccer players aged 13 to 17 years. *PloS One*. **10** (12). 10.1371/journal.pone.0144264 (2015). e0144264. 10.1371/journal.pone.0144580 PMC4691195 26657073

[44] Scharfen, H. E. & Memmert, D. The relationship between cognitive functions and sport-specific motor skills in elite youth soccer players. *Front. Psychol.* **10**, 817. 10.3389/fpsyg.2019.00817 (2019). 31105611 10.3389/fpsyg.2019.00817 PMC6494938

[45] Sitaram, R. et al. Closed-loop brain training: the science of neurofeedback. *Nat. Rev. Neurosci.* **18** (2), 86–100. 10.1038/nrn.2016.164 (2017). 28003656 10.1038/nrn.2016.164

[46] Luck, S. J. & Kappenman, E. S. (eds) *The Oxford Handbook of event-related Potential Components* (Oxford University Press, 2011).

[47] Pascual-Leone, A., Amedi, A., Fregni, F. & Merabet, L. B. The plastic human brain cortex. *Annu. Rev. Neurosci.* **28**, 377–401. 10.1146/annurev.neuro.27.070203.144216 (2005). 16022601 10.1146/annurev.neuro.27.070203.144216

[48] Yu, S., Lee, K., Park, H. & Yoon, B. The effect of EEG neurofeedback training on sport performance: A systematic review and meta-analysis. *Scand. J. Med. Sci. Sports*. **35** (1), e70055. 10.1111/sms.70055 (2025). 40270441 10.1111/sms.70055 PMC12019780

[49] Cramer, S. C. et al. Harnessing neuroplasticity for clinical applications. *Brain* **134** (6), 1591–1609. 10.1093/brain/awr039 (2011). 21482550 10.1093/brain/awr039 PMC3102236

[50] Sterman, M. B. & Friar, L. Suppression of seizures in an epileptic following sensorimotor EEG feedback training. *Electroencephalogr. Clin. Neurophysiol.* **33** (1), 89–95. 10.1016/0013-4694(72)90028-4 (1972). 4113278 10.1016/0013-4694(72)90028-4

[51] Kwon, H., Maeng, H. & Chung, J. Development of an ICT-Based exergame program for children with developmental disabilities. *J. Clin. Med.* **11** (19), 5890. 10.3390/jcm11195890 (2022). 36233757 10.3390/jcm11195890 PMC9572951

[52] Formiglio, E. et al. The effects of multiple factors on post-activation potentiation and performance enhancement: a narrative review. *Acta Kinesiologica*. **18** (1), 75–91 (2024).

[53] Gadea-Uribarri, H. et al. Concordance of a new IMU in different small-sided games and real game tasks in indoor sports: A new IMU valid for real game situations in indoor sports. *Acta Kinesiologica*. **18** (3), 53–60 (2024).

[54] Kuliś, S., Kłobuchowski, W., Skorulski, M., Pietraszewski, P. & Callegari, B. Upper limb tremor variability in elite sport dancers: the influence of competitive simulation. *Biomedical Hum. Kinetics*. **17** (1), 219–228. 10.2478/bhk-2025-0021 (2025).

[55] Kuliś, S., Pietraszewski, P. & Callegari, B. Characteristics of Post-Exercise lower limb muscle tremor among speed skaters. *Sensors* **25** (14), 4301. 10.3390/s25144301 (2025). 40732429 10.3390/s25144301 PMC12298239

